# TIPIT: A randomised controlled trial of thyroxine in preterm infants under 28 weeks' gestation

**DOI:** 10.1186/1745-6215-9-17

**Published:** 2008-03-26

**Authors:** Sze M Ng, Mark A Turner, Carrol Gamble, Mohammed Didi, Suresh Victor, Alan M Weindling

**Affiliations:** 1 School of Reproductive and Developmental Medicine, University of Liverpool, Liverpool, UK; 2 Centre for Medical Statistics and Health Evaluation, University of Liverpool, Liverpool, UK; 3 Department of Endocrinology, Royal Liverpool Children's Hospital, Liverpool, UK; 4 Maternal and Fetal Health Research Group, School of Clinical and Laboratory Sciences Faculty of Medical and Human Sciences, University of Manchester, Manchester, UK

## Abstract

**Background:**

Infants born at extreme prematurity (below 28 weeks' gestation) are at high risk of developmental disability. A major risk factor for disability is having a low level of thyroid hormone which is recognised to be a frequent phenomenon in these infants. At present it is unclear whether low levels of thyroid hormone are a cause of disability, or a consequence of concurrent adversity.

**Methods:**

We propose an explanatory multi-centre double blind randomised controlled trial of thyroid hormone supplementation in babies born below 28 weeks' gestation. All infants will receive either levothyroxine or placebo until 32 weeks' corrected gestational age. The primary outcome will be brain growth. This will be assessed by the width of the sub-arachnoid space measured using cranial ultrasound and head circumference at 36 weeks' corrected gestational. The secondary outcomes will be (a) thyroid hormone concentrations measured at increasing postnatal age, (b) status of the hypothalamic pituitary axis, (c) auxological data between birth and 36 weeks' corrected gestational age, (d) thyroid gland volume, (e) volumes of brain structures (measured by magnetic resonance imaging), (f) determination of the extent of myelination and white matter integrity (measured by diffusion weighted MRI) and brain vessel morphology (measured by magnetic resonance angiography) at expected date of delivery and (g) markers of morbidity including duration of mechanical ventilation and chronic lung disease.

We will also examine how activity of the hypothalamic-pituitary-adrenal axis modulates the effects of thyroid supplementation. This will contribute to decisions about which confounding variables to assess in large-scale studies.

**Trial registration:**

Current Controlled Trials ISRCTN89493983

## Background

In the immediate postnatal period of babies born at term, hypothyroidism causes neurodevelopmental disability[1,2]. This disability can be prevented by giving supplementary thyroid hormone[3,4]. Infants born at extreme prematurity (i.e. before 28 weeks' gestation) are at high risk of neurodevelopmental disability, particularly if they are hypothyroid [4-6]. Some, but not all, forms of thyroid supplementation appear to reduce the incidence of poor neurodevelopmental outcome in infants born at extreme prematurity [7-9].

### Hypothyroidism in early infancy causes neurodevelopmental disability which can be prevented by giving supplementary thyroid hormone

In congenital hypothyroidism (CH) early treatment with thyroid replacement reduces severe developmental deficits[1]. Even mild thyroid hormone insufficiency can produce measurable, but preventable, deficits in neuropsychological functions[3,4]. Magnetic resonance imaging (MRI) and spectroscopy (MRS) have documented brain metabolic changes in untreated CH. The reversal of these abnormalities following replacement therapy is thought to be due to improved myelination[10]. This suggests an important role for thyroid hormone in the developing brain in the early postnatal period.

### Infants born before 28 weeks of gestation are at high risk of disability

Advances in neonatal medicine have resulted in an increase in survival of preterm infants below 28 weeks' gestation. Infants born extremely prematurely at this low gestation are at particular risk of neurological abnormalities [11-13]. The normal development of the brains of these infants is at high risk of disruption with consequent acquired damage especially to the white matter because of multiple factors[14]. These are thought to include hypoxic ischaemia, free radical injury because of low concentrations of antioxidants, undernutrition and sepsis[15]. This period in brain development is important because neuronal migration has only just finished (at about 20 weeks) and synaptogenesis is occurring and therefore the cellular milieu is critical[16].

Brain MRI studies of survivors of low birth weight, who have subtle cognitive abnormalities have been shown to have diminished volumes of the caudate nucleus and hippocampus and these findings have been correlated with lower IQ, learning disorder and attention deficit[17,18]. Recent brain MRI studies of preterm infants also show that quantitative cerebral structural abnormalities are related to the degree of immaturity at birth and are followed by abnormal neurodevelopmental outcome[14,19]. Preterm infants assessed at term show reduced myelination when compared to infants born at term[14].

### Evidence of hypothyroidism is common among infants born before 28 weeks of gestation and is associated with an increased risk of disability

Transient hypothyroxinaemia of prematurity as characterised by low blood thyroxine (T4) concentrations with normal or mildly elevated plasma thyroid stimulating hormone (TSH) concentrations is common in infants under 30 weeks' gestation at birth, occurring in up to 69%[9,20-23]. The proportion of preterm infants having hypothyroxinaemia increases with decreasing gestational age[22,24]. A recent large collaborative Scottish study has shown that an extremely premature group of infants of 23–27 weeks' gestation appeared distinctive and were hypothyroxinaemic relative to other groups[20]. The postnatal surge of T4 concentrations described in the term infants was less marked in infants of 31–34 weeks gestation, absent in infants of 28–30 weeks and reversed in infants of 23–27 weeks who had low plasma T4 concentrations.

Low blood thyroid hormone concentrations in the first few weeks of life in preterm infants have been linked with poor neurodevelopment[5,6]. Reuss et al[25] (n = 463) found a 4-fold increased risk of disabling cerebral palsy at 2 years of age associated with hypothyroxinaemia in infants born below 33 weeks' gestation. Den Ouden et al[26] (n = 563) found a higher incidence of neurologic dysfunction at 5 and 9 years of age in association with lower thyroxine concentrations even after adjustments for other perinatal factors in preterm infants born below 32 weeks'. Paul et al[27] found increased mortality and increased incidence of intraventricular haemorrhage in preterm infants with abnormal thyroid function, whilst Leviton et al[26] reported white matter changes predictive of abnormal neurological outcome on cranial ultrasound of neonates with low T4 blood concentrations. In addition, published data have documented a relationship between maternal thyroid deficiency during pregnancy and problems with neuropsychological development of the offspring[28]. However, the questions of whether this hypothyroidism reflects the adversity leading to brain injury, or whether hypothyroidism is a preventable cause of brain injury can only be answered by randomised controlled clinical trials.

### Thyroid supplementation to extreme preterm infants may reduce the risk of disability

A randomised controlled trial of T4 supplementation by Van Wassanaer et al[8,21] showed an improved Bayley Mental Development Index (MDI) and Psychomotor Development Index (PDI) at 2 years among infants born at extreme prematurity who had received T4 supplementation. However, the babies who appeared to benefit were not the primary target population in this study and the positive result was only evident after subgroup analysis. In the whole sample there was no benefit to long term outcome. The reliability of that trial was limited by a small sample size. In other studies, thyroid hormone replacement has been given to preterm infants with either beneficial effects or no apparent effects[7,9,21,29,30]. However, the studies which have been done are of small sample size and have not shown rigorous proof of efficacy of such a treatment.

### Previous studies of thyroid replacement therapy in preterm infants

A Cochrane review of thyroid hormone supplementation in premature infants found that none of the available trials provide good evidence for the value of thyroid hormone supplementation in preterm infants[31]. The results of the meta-analysis found no significant difference in overall mortality. Meta-analysis of two studies[7,8] found no significant difference in Bayley MDI or PDI performed at 7–12 months, death or IQ score at 5.7 years of age. All studies were of small size with the largest by van Wassanaer[8], which enrolled 200 infants under 30 weeks' gestation. However, the sample size for this post hoc subgroup analysis of infants with gestational age below 27 weeks who benefited from the intervention was small comprising just 46 infants (19 in the treatment arm and 27 in the placebo arm). The review concluded that future trials of sufficient size needed to be undertaken to detect the clinically important differences in neurodevelopmental outcome, particularly enrolling the infants most likely to benefit from thyroid hormone therapy such as extremely preterm infants.

The conclusion from these results is that there is biologically plausible evidence that T4 supplementation for very immature babies may improve their neurological outcome, but that more mature babies are not likely to benefit from this intervention. Studies of triiodothyronine (T3) supplementation[22,30] have not provided evidence for improved outcome. Animal studies have shown that the postnatal development of brain structures in rats was entirely dependent on local generation of T3 from T4 by type 2 iodothyronine deiodinase[32,33]. This suggests that normal neurodevelopment requires appropriate T4 supplementation and not T3 supplementation. T3 supplementation appeared to raise plasma free T3 and total T3 concentrations but suppress T4 concentrations[34,35]. T4 supplementation has been shown to suppress T3 concentrations but raise total and free T4 concentrations (FT4)[7,8]. Van Wassanaer et al examined different T4 dosage schemes and concluded that 8 mcg/kg birthweight/day increased T4 and FT4 concentrations most appropriately and prevented transient hypothyroxinaemia during the 6 weeks' treatment[35]. Van Wassanaer also showed that high longitudinal FT4 concentrations were not associated with worse outcome, while low FT4 concentrations were associated with worse neurodevelopmental outcome at 2 and 5 years of age[35]. In the light of these findings, a trial of T4 supplementation is a strong candidate for intervention.

An important practical issue for intervention studies is the need to classify the thyroid status of individual infants. The blood concentrations used in this classification will determine recruitment to the study and the response to monitoring during the intervention. At present, it remains unclear what cut-off point for T4 or FT4 concentrations constitutes hyperthyroxinaemia or hypothyroxinaemia in these preterm infants, particularly in the weeks just after birth. Normative values for thyroid hormones in cord blood for each gestational age have been published[20,24]. It has been suggested that these values can provide normative values in the postnatal period by relating concentrations at a particular corrected gestational age to those of infants born at the same gestational age[20]. However, the implications of this strategy have not been explored in treated infants. We will be able to model the implications of this strategy using the results of this trial.

### The associations of transient hypothyroidism have not been studied among infants born at extreme prematurity

Following birth, thyroid status changes and these changes are influenced by gestational age at birth. The consequences of these changes have not been reported. Longitudinal measures of thyroid status and neonatal progress will be made during this trial and will allow us to describe how thyroid status in the weeks after birth at extreme prematurity is related to neonatal course.

### Thyroid status may affect other organ systems in ways that affect the design of future trials

Thyroid hormones alter metabolic rate[2,28]. Nutritional and growth implications of hypothyroidism have not been studied. Thyroid supplementation could increase nutritional requirements and reduce extrauterine growth. To date, there is little evidence of this occurring among infants born at extreme prematurity. Given that neurodevelopmental outcome may be confounded by the social environment after discharge[13,36], it will be important to include neuroradiological markers of brain development as an intermediate outcome. However, although thyroid hormones are involved in brain development, particularly in myelination[10,37,38], there is a paucity of published data concerning the relationship between thyroid hormone status and brain development in preterm infants. The lack of information about how thyroid status relates to brain size and body growth will hinder clinical trials of thyroid supplementation.

### Other factors may be relevant to the effects of thyroid supplementation on outcome

Factors which may contribute to abnormal thyroid function in very preterm infants include: immaturity of the thyroid gland, immaturity of the hypothalamic-pituitary-thyroid axis, disruption of maternal transfer of T4 through the placenta, relative immaturity of type 1 deiodothyronine monodeiodinase enzyme systems and reduction in thyroid binding globulin (TBG) due to hepatic immaturity[2,23]. Iodine is required for renewal of intra-thyroidal T4, but the concentrations of iodine in the thyroid gland of preterm infants are low[39,40]. This appears to be related to maternal iodine status and effect of inadequate iodine supplementation in parenteral nutrition for preterm infants [39-41].

Abnormal thyroid size is often found in disease states, e.g. goitre[2]. Thyroid size may therefore be a useful marker of thyroid status. However, the only normative study of thyroid size in term infants was limited to 100 infants and did not examine infants born at extreme prematurity[42].

Blood cortisol concentrations decrease as blood T4 concentrations increase with advancing gestational age[34,43]. These observations suggest that there may be an interaction between the cortisol and thyroid systems in preterm infants. Since both hormones have been implicated in early brain development[44,45], this interaction could contribute to the poor neurological outcome of infants born extremely premature. Activity of the hypothalamic-pituitary-adrenal (HPA) axis is central to normal fetal development and neonatal transition[46,47]. Extremely premature infants have low basal blood cortisol concentrations and inadequate stimulated cortisol responses to adrenocorticotrophic hormone in the early neonatal period[44,47]. Cortisol is important for survival during illness and cortisol concentrations are expected to increase during significant stress. In ill adults with low baseline and stimulated cortisol concentrations, mortality is higher[44]. In the preterm infant population, the physiological significance of lower blood cortisol values remains unclear[46,47]. Most studies have not consistently correlated blood cortisol concentrations to physiological markers of cortisol effect and outcome in preterm infants. In a recent study, the HPA axis was found to be unresponsive to standard stimulation tests until 11 days of age in preterm baboons indicating HPA axis immaturity[48]. Activation of the HPA axis and increased blood cortisol concentrations may be essential to respond to the many stressors of extra-uterine existence in the extreme preterm infants. This transient inability of the adrenals to maintain cortisol homoeostasis in premature newborns in the immediate postnatal period has been described as transient adrenocortical insufficiency of prematurity[44].

Valerio et al[30] looked at the effect of thyroid hormone supplementation on blood cortisol concentrations in babies born below 28 weeks GA. The sample size was small with only 31 patients. No significant difference was noted in cortisol concentrations and mortality between the groups. The results of the study suggest that hypothyroxinaemia was not involved in the development of hypocortisolism in preterm infants. However, the medium-term effects of the relationships between these two endocrine systems have not been studied. Further understanding of their roles in the outcome of preterm infants is needed.

### Possible negative effects of giving T4

Thyroxine is infrequently given to extremely premature babies. The only adverse effect that might be predicted is based on clinical experience of giving the drug to more mature patients, namely a persistent tachycardia. The drug may increase metabolic rate. In the critical paper by van Wassanaer et al[8], there was no difference in neurodevelopmental outcome as a result of T4 supplementation. However, a *post hoc *subgroup analysis showed that more mature babies who were given T4 supplementation had a worse neurodevelopmental outcome than those who were unsupplemented. The mechanism for this possible effect is not known.

### Levothyroxine pharmacokinetics

There has been no previous work in this patient group on the circulating blood concentrations of thyroid hormones following the administration of levothyroxine. Establishing whether each infant has similar circulating blood concentrations will be an important aspect of the explanatory RCT. We will use population pharmacokinetic techniques. These will allow us to derive information about circulating thyroid hormone concentrations using measurements done on blood samples taken during routine clinical practice.

In summary, the literature indicates that several areas of uncertainty will hamper randomised controlled studies of interventions for hypothyroidism in extremely preterm infants. These areas include: the optimal timing of monitoring, whether it is appropriate to use reference ranges based on cord blood values, the effect of cortisol status on thyroid function and the relationship between thyroid status and HPA axis. None of these issues can be clarified unless infants who receive thyroid supplementation are prospectively compared to infants who do not receive thyroid supplementation.

In the light of the issues outlined above, an explanatory[49] randomised controlled trial of thyroid supplementation is proposed in infants below 28 weeks' gestation, with an assessment of its effect on short term explanatory outcomes. This study will markedly enhance the design and interpretation of future large-scale studies aimed at determining the effect of such supplementation on long term neurodevelopmental outcome.

There is therefore an urgent need for a larger scale study to discover whether thyroid supplementation benefits these very premature babies. This proposed study will examine the effects of one commonly used form of thyroid supplementation on key intermediate outcomes in these infants. We will also extend previous work in this area by exploring perinatal factors which could modulate the effects of thyroid supplementation

## Aims of the study

To determine how treatment with levothyroxine (LT-4) postnatally until 32 weeks corrected gestational age (CGA) modulates brain size and development, the hypothalamic-pituitary-adrenal axis (HPA) and somatic growth in a randomised double blind placebo-controlled trial.

### Objectives

#### Primary Outcome

• Effect of levothyroxine therapy on brain size as assessed by the width of the subarachnoid space [54] measured at 36 weeks CGA using cranial ultrasound and serial head circumference as an index of brain weight[50,51].

#### Secondary Outcomes

(a) Serial head circumference as an index of brain weight

(b) Longitudinal thyroid hormone concentrations between birth and expected date of delivery;

(c) Cortisol hormone status between birth and expected date of delivery

(d) Auxological data at pre-randomisation, day 14, day 21, day 28 and at 36 weeks' CGA

(e) Thyroid gland size measured by ultrasound imaging

(f) Mortality

(g) The volumes of key brain structures, whole brain volume, extent of myelination of cerebral white matter and white matter integrity and brain vessel morphology using brain MRI examinations at term equivalent

(h) Duration of mechanical ventilation/oxygen requirement

(i) Chronic lung disease requiring home oxygen at discharge

## Methods

### Trial Design

A multicentre double blind randomised placebo controlled trial of levothyroxine given postnatally to infants born under 28 weeks' gestation until 32 weeks' CGA. Parents, care providers and outcome assessors will be blind to the allocation of placebo and levothyroxine.

### Inclusion Criteria

a) Infants with gestational age under 28 weeks at birth*

b) Recruitment within five days of birth

c) Single or multiple births

*The assessment of gestational age will be based on the working estimate available in the obstetric notes at the time of delivery (corrected gestational age).

### Exclusion Criteria

a) Infants born to mother with known thyroid disease or on antithyroid medications during pregnancy will be excluded.

b) Infants born to mother who are on amiodarone during pregnancy will be excluded.

c) Infants diagnosed with major congenital or chromosomal abnormalities known to affect thyroid function or brain development will be excluded.

d) Maternal death during or within 5 days after childbirth.

### Randomisation

Once all inclusion and exclusion criteria are met and informed consent has been obtained, infants will be included in the study and allocated a randomisation number. The randomisation lists will be computer generated with random variable block lengths using the statistical package STATA. The randomisation list will be stratified by centre, and gestational age at birth (under 25 weeks' gestation, 25–26 weeks' gestation, 27–27^+6 ^weeks' gestation). Multiple births will follow the randomisation process as per singleton births: each sibling in a multiple birth will receive the same treatment allocation.

Concealment of allocation will be provided by pharmacy departments of the respective hospitals. The randomisation list will be stored in a locked filing cabinet in the pharmacy Departments of each hospital and access will be limited to the pharmacist involved in the study.

#### Blinding and Unblinding

Parents, investigators, clinicians involved in patient care and individuals assessing and analysing study end-points will all be blinded to the allocated study treatments. Levothyroxine and its placebo will be presented identically (see below). In the event that unblinding is necessary, there will be 24 hour access for unblinding and an Unblinding Case Report Form will be completed. In most cases, stopping the 'treatment' should be sufficient without the need for unblinding. Before unblinding is done, the case will be discussed with the Chief Investigator or in his absence a delegated member of the Trial Management Group.

### Treatment Regimen and Dosage

We will use two forms of the active medication: intravenous levothyroxine (tradename: Levothyroid) and oral levothyroxine solution (tradename: Evotrox) with corresponding placebos during the study period.

In the initial phase, infants will receive either intravenous Levothyroid or placebo (5% dextrose) within the first 5 days after birth. The treatment regimen will follow the dosage guidelines determined by previous studies[8,52] at 8 mcg/kg birthweight/day single daily dose.

In the next phase, once enteral feeds are fully established, oral solution Evotrox or placebo (carrier solution without active drug) will be given at 8 mcg/kg birthweight/day single daily dose until the baby reaches 32 weeks CGA.

### Study Medication

The intravenous levothyroxine (Levothyroid) medication is a powder and is dissolved in 5% dextrose and albumin and aliquots are put into syringes aseptically by the Pharmacy Department at the Royal Liverpool and Broadgreen University Hospital. Albumin is added to the reconstitution based on previous similar work done by van Wassanaer et al[8]. The addition of a small amount of albumin results in a more stable solution with no decomposition products and little possibility of losses due to adsorbtion/absorption of levothyroxine into the syringe. The addition of albumin therefore allows for the availability of free thyroxine. Stability testing for the levothyroxine was performed by Quality Control North West.

Levothyroid is manufactured in Spain by Aventis Pharma, Marketing Authorisation 971622. It is imported by IDIS Ltd and prepared under EU Good Manufacturing Practice at Royal Liverpool University. Placebo will be an equal volume of 5% dextrose presented in an identical manner to the Levothyroid.

Evotrox is an oral solution and is manufactured in the United Kingdom by Kappin Ltd, Marketing Authorisation PL20249/0007. The placebo form is prepared by the Pharmaceutical company under the terms of their Marketing Authorisation as the identical carrier substance without the active ingredient. Dispensing and blinding is by the Pharmacy Departments at recruiting centres.

### Blood and Saliva Sampling

Infants: All infants will undergo blood investigations. Each sample will require approximately 0.5 ml of blood and this is arranged to coincide with routine blood tests obtained during the clinical care of the infant at the neonatal unit

TT4 (total thyroxine), FT4 (free T4), TT3 (total T3), FT3 (free T3), TSH (thyroid stimulating hormone) blood concentrations will be measured before treatment begins (baseline) and on Day 14, Day 21, Day 28 and 36 weeks (corrected age).

A subset population of infants may have more frequent blood tests for thyroid function to test the bioavailability and pharmacokinetics of the drug. The blood test will be done at different times of the day on different babies and this is arranged to coincide with routine blood tests obtained during the clinical care of the infant at the neonatal unit. Consent will be obtained separately from the parents of these infants. Since treatment allocation will be concealed, some of these infants will be receiving placebo. Comparison of drug concentrations between the two treatment groups will allow us to take account of background variation in blood thyroid concentrations.

All infants will have saliva collected from the mouth (100 microlitres) at three separate times of the day before treatment begins (baseline), on day 14, Day 28 and 36 weeks' CGA.

Early morning cortisol and ACTH concentrations will be obtained before treatment begins (baseline), on Day 14, Day 28 and 36 weeks CGA.

Mother of infant(s) enrolled: 5 ml of blood for TT4, FT4, TT3, FT3, TSH, thyroid peroxidase antibody (TPO), cortisol and thyrotropin receptor antibody (TRAB) will be obtained as soon as possible from mothers of infants who are enrolled to the study. These results will be reviewed by the study team and abnormal results will be referred to a Consultant Endocrinologist, who will liaise with the mother's Obstetrician about any follow-up that is required.

### Growth and Auxology

All infants will be measured for weight, lower leg length (using knemometry) and occipito-frontal circumference before commencing treatment (baseline), day 14, day 21, day 28 and at 36 weeks' CGA.

### Brain Imaging

Cranial ultrasounds will be performed according to the Neonatal Unit's protocol. There will be study scans at 36 weeks' CGA and routine images will be supplemented by measurements of width of the subarachnoid space.

MRI examination of the brain will be performed at term equivalence in infants for whom separate informed parental consent will be obtained, at the MRI department of the Royal Liverpool Children's Hospital, Liverpool or Wellcome Trust Clinical Research Facility, Manchester. Data acquisition is not expected to exceed 40 minutes. No sedation will be used, as infants will be imaged during natural sleep after having been fed. Careful infant positioning by the radiographer and temperature maintenance will ensure their comfort while being scanned.

### Thyroid Imaging

We will also measure thyroid gland size using ultrasound at 36 weeks' CGA. These data will be compared to published normative data for measurements of thyroid size in utero and [53]in term infants[42]. In conjunction with detailed characterisation of thyroid hormone status, this will provide a novel insight into how prematurity affects the thyroid axis.

## Summary of Investigations and Procedures

### Processing of Blood and Saliva Samples

**Figure 1:**
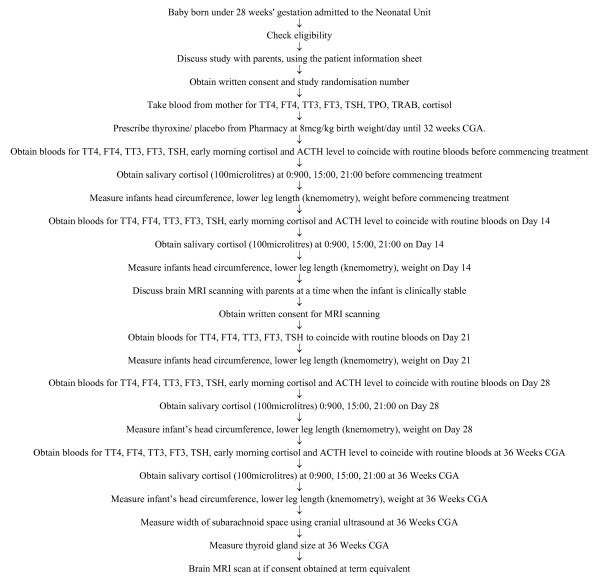
Patient Flow During Trial.

All blood samples will be processed according to standard practice and sent to the laboratories at the Royal Liverpool Children's Hospital (Alder Hey). Saliva samples will be sent to University Department Laboratory located in the Liverpool Women's Hospital for processing (Figure 1).

### Informed Consent

The parent(s)/guardian(s) of each potentially eligible patient will be informed of the study's objectives and overall requirements after birth when the baby has achieved respiratory and haemodynamic stability. The Investigator will explain the study fully using the Patient Information Leaflet. The parent/guardian will then be given at least 12 hours to consider the study. If a parent/guardian is willing for the patient to participate in the study, he/she will be requested to give written consent. The Informed Consent Form will be signed and personally dated by both a parent/guardian and the investigator. A copy of the signed form will be provided to the parent(s)/guardian(s), another copy will be placed in they patient record, and the original retained with the research record. Nursing staff may be involved in describing the trial to the patient and his/her parent(s)/guardian(s).

A similar procedure will be followed for the elements of the study relating to MRI scans and population pharmacokinetics. An investigator will introduce the MRI scan/population pharmacokinetic study, leave the specific information leaflet and return after at least 12 hours.

### Data Management

Data will be collected on a case report form (CRF) starting at the time of recruitment. Data will be entered anonymously on to a password-protected database which will be stored in a secure office. All CRF hard copies will be duplicated and will be stored in a locked filing cabinet within the same office. Access to the database will be restricted to the investigators.

In accordance with R&D policy at LWH, the notes of all participants will be marked with a sticker to indicate study participation. A tag will be used to identify electronic records of study participants. All clinical records of study participants will be retained for 20 years. All paper and electronic records relating to the study will be retained for 20 years.

### Subject Withdrawal

Patients may be withdrawn from treatment for any of the following reasons:

a) Parent/legal representative withdraws consent.

b) Unacceptable toxicity (a potential unacceptable toxicity is defined as sustained tachycardia i.e. heart rate greater than 220 beats per minute for more than 30 minutes in a 60 minute period, in the presence of a high thyroid hormone blood concentration)

c) Intercurrent illness preventing further treatment.

d) Development of serious disease.

e) Any change in the patient's condition that justifies the discontinuation of treatment in the clinician's opinion.

If a parent/legal representative wishes to withdraw an infant from trial treatment, centres will document the reason and explain the importance of remaining on trial follow-up and, if willing, to continue to have data collected as per trial schedule, or, failing this, to attend clinic and allow routine follow-up data to be used for trial purposes. Generally, follow-up will continue unless the patient explicitly withdraws consent for follow-up. Following withdrawal from trial treatment, patients will be treated according to usual local clinical practice.

The parents/legal guardians of patients who voluntarily withdraw from the trial have previously consented to follow-up in the trial. Data up to the time of withdrawal will be included in the trial if anonymised. If the parents/legal guardians explicitly state their wish not to contribute further data to the study, the investigators will be informed and a withdrawal CRF will be completed.

## Statistical Consideration

### Sample Size Calculation

A sample size of 64 in each group will have 80% power to detect a difference between the means of the thyroxine and placebo groups of 0.67 mm (0.5*SD) for the outcome subarachnoid space at 36 weeks' CGA. This assumes that the common standard deviation is 1.3 mm and analysis is based on using a two group t-test with a 0.05 two-sided significance level. The value for the SD is taken from Armstrong et al[54] who report subarachnoid space as an indirect method of brain growth in preterm infants.

To ensure the appropriateness of the SD used in our calculations we will run an internal pilot after the first 30 infants have been randomised. We will compare the SD of these 30 infants with the standard deviation of Armstrong et al[54]. An internal pilot[49] can be used to increase but not decrease the size of the study. Assuming the 20% loss to follow up that we have experienced in an ongoing trial on the unit, we aim to recruit and randomise 150 infants with gestation below 28 weeks'.

#### Analysis

Final analysis of the primary and secondary outcome measures will be carried out at study closure defined as the date of database lock. The principle of intention to treat analysis will be performed blind to treatment allocation.

Longitudinal data will be analysed using mixed models with survival outcomes analysed using Kaplan-Meier curves with log-rank tests and Cox Proportional Hazard models. The potential for jointly modelling longitudinal data with survival outcomes will be explored[50]. The use of these regression models will allow us to consider the relationships between thyroid status and factors that could modulate the effect of thyroid supplementation. A separate statistical analysis plan will be developed prior to the internal pilot.

A secondary per protocol analysis will also be considered.

## Adverse Event Reporting and Safety Evaluations

### Definitions

#### Adverse event (AE)

Any untoward medical occurrence in a subject recruited to a clinical trial, including occurrences which are not necessarily caused by or related with the treatment. An adverse event can be any unfavourable and unintended sign or symptom associated with the use of a medicinal product whether or not related to that product.

#### Adverse reaction (AR)

Any untoward and unintended response in a subject to an investigational medicinal product which is related to any dose administered to that subject.

#### Serious adverse event (SAE), serious adverse reaction (SAR) or unexpected serious adverse reaction

Any adverse event, adverse reaction or unexpected adverse reaction, respectively, that

a) results in death,

b) is life-threatening,

c) requires hospitalisation or prolongation of existing hospitalisation,

d) results in persistent or significant disability or incapacity, or

e) consists of a congenital anomaly or birth defect.

### Expected Serious Adverse Events

Extremely premature babies below 28 weeks' gestation constitute a high risk population: the following are serious adverse events and incidence rates which could be reasonably expected for this group of babies during the course of the study (Table 1).

**Table 1:** List of Expected Serious Adverse events

Adverse event	Estimated incidence
a) death	20%*
b) necrotising enterocolitis or focal intestinal perforation diagnosed on clinical grounds, or at surgery	15%*
c) intracranial abnormality (parenchymal haemorrhage or focal white matter damage) on cranial ultrasound	15%*
d) requirement for supplementary oxygen 28 days after birth	55%*
e) patent ductus arteriosus requiring medical or surgical management	25%*
f) retinal surgery for retinopathy of prematurity	5%*
g) sustained tachycardia (greater than 220 beats per minute for more than 30 minutes in a 60 minute period)	20%**
h) pulmonary haemorrhage	5%**
i) persistent weight loss after 14 days after birth in the absence of infection.	10%**

* based on incidence of these complications at Liverpool Women's Hospital

** based on incidences estimated by the investigators

### Reporting Serious Adverse Events

All expected Serious Adverse Events (SAE) (Table 1) will be recorded in the case report forms (CRF). All deaths or suspected overdoses will be reported using the SAE report forms (See Appendix) to the Chief Investigator (CI) within 24 hours and will be reviewed by the DMEC at regular intervals throughout the trial. The trial sponsor will collate and submit annual safety reports to MHRA and ethics committee. However, the MHRA, the trial sponsor and the chair of the MREC may also be informed about severe adverse events by the DMEC chair if considered appropriate.

A standard reporting procedure will be provided on the reverse side of all SAE forms. A Trial Standard Operating Procedure with detailed information on reporting procedures will also be kept in site files at each 'trial station' in each centre. Copies of all correspondence relating to reporting will be maintained in the Investigator's files.

All adverse events whether or not they are attributable to the study intervention will be reported on case report forms. These will be reviewed by the investigators monthly to determine if there is reasonable suspected causal relationship to the intervention. If there is evidence that there may be a novel causal relationship with the intervention, then the procedure for expedited reporting of suspected unexpected serious adverse reactions will be followed (section 5.4 below). All adverse events will be followed until satisfactory resolution or until the investigator responsible for the care of the participant deems the event to be chronic or the patient to be stable.

### Suspected Unexpected Serious Adverse Reactions

Given the vulnerability of these infants, the Chief Investigator and the Principal Investigators will review all serious adverse events in order to detect Suspected Unexpected Serious Reactions (SUSARs), involving other members of the Trial Management Group as appropriate. The procedure for identifying and reporting Suspected Unexpected Serious Adverse Reactions will be as follows.

All deaths or overdoses will be reviewed by the Chief Investigator (CI) or in his absence another member of the Trial Management Group. For each death the CI, or his deputy, will make a judgement about whether the nature or severity of the circumstances surrounding the death is consistent with adverse drug reactions associated with levothyroxine. Other members of the Trial Management Group who are available at the time may contribute to a discussion prior to the judgement. The judgement will take place within 24 hours of the notification of a death. If the nature or severity of the events leading up to the death are considered to be related to the study medication but inconsistent with what is known about levothyroxine, the allocation concealment for the affected infant will be broken by the sponsor. In the event of a death occurring in association with events that are not consistent with the information contained in this protocol and the infant was allocated to levothyroxine, the event will be labelled as a Suspected Unexpected Serious Adverse Reaction (SUSAR). If an overdose is thought to have led to a serious adverse event not listed in section 5.2 then the event will be labelled as a SUSAR. All events labelled as SUSARs will be reported to the sponsor within 24 hours of the decision being made. All SUSARs will be reported to the competent regulatory authority (MHRA) with a copy to the Sponsor as soon as possible but not later than 7 working days.

The Trial Management Group will meet monthly in order to review all other Serious Adverse Events. The Group will consider whether each SAE is consistent with the information contained in this protocol about the nature and severity of adverse drug reactions associated with levothyroxine (including literature cited in the protocol). The CI or his deputy will make a judgement about whether the nature or severity of the circumstances surrounding the death is consistent with the information contained in this protocol about the nature and severity of adverse drug reactions associated with levothyroxine. If the nature or severity of the SAE is judged to be inconsistent with the information contained in this protocol the allocation concealment for the affected infant will be broken. In the event that a SAE occurs in association with events that are not consistent with the information contained in this protocol and the infant was allocated to levothyroxine, the event will be labelled as a Suspected Unexpected Serious Adverse Reaction (SUSAR) and reported as above.

## Trial Oversight

### Data Monitoring and Ethics Committee (DMEC)

An independent Data Monitoring and Ethics Committee (DMEC) has been formed. During the period of recruitment, interim summaries of results will be supplied, in strictest confidence, to the DMEC by the trial statistician. At its first meeting the DMEC will determine the frequency of its meetings and the data to be supplied for the meetings. Meetings of the full committee will be arranged periodically as agreed by the full DMEC at the first meeting. In the light of interim data and emerging evidence from other studies, the DMEC will inform the Trial Steering Committee if, in their view, there is proof beyond reasonable doubt that the data indicate that any part of the protocol is indicated or contra-indicated either for all infants or for a particular subgroup of trial participants.

The decision to inform the steering committee will be, in part, based on statistical considerations. Appropriate criteria for the 'proof beyond reasonable doubt' cannot be specified precisely, but a difference of at least 3 standard deviations of a major endpoint may be needed to justify halting or modifying the study prematurely. If a 2–5 fold increase, depending on the event, is seen in any of the expected serious adverse events across both groups, then an emergency DMEC meeting will be called. However, discretion will used around the cut-off points based on discussion with the Trial Management Group members.

### Trial Steering Committee (TSC)

A trial steering committee will be set up which will have overall supervision of the trial. It will meet (generally by telephone conference) prior to commencement of the trial and then at least 6 monthly until completion. A meeting of the TSC will be held within a month of every DMEC meeting to consider their recommendations.

### Significance of the Trial

The optimal way to manage transient hypothyroidism in preterm infants under 28 weeks' gestation is unclear. In part, this is because infants born at extreme prematurity have different physiology from older children, preterm delivery leads to some unique complications and infants undergo striking maturational changes during their stay on the intensive care unit. Thus, studies that test whether manipulation of the thyroid system alters outcome need to be based on a refined understanding of how thyroid supplementation relates to premature birth, its complications and subsequent development. Recent guidelines from the National Institute for Clinical Excellence (NICE) emphasise this need for including such children in rigorous studies of interventions rather than treating them on an *ad hoc *basis. To date, other studies have not reported serious harm as a consequence of T4 therapy in preterm infants. The role of thyroid hormone therapy in preterm infants under 28 weeks gestation will be established or refuted as a consequence of this trial. The information acquired will be of interest to neonatologists, obstetricians, paediatric endocrinologists and a wide circle of general paediatricians in their management of preterm infants. This will help to ensure those who need treatment receive it, and those who do not need treatment will not receive inappropriate therapy.

This study will yield novel data about how supplementation with thyroid hormone of an extreme preterm infant modulates the hypothalamic-pituitary-adrenal axis, somatic growth and brain size at term. It is an explanatory trial designed to provide data that will provide essential underpinning to pragmatic trials of treatment efficacy by clarifying the factors that need to be accounted for in large-scale studies and providing estimates of variability in key confounders.

### Ethical issues

There is no evidence that T4 supplementation causes harm in our target population. The most likely negative effect of T4 supplementation (persistent tachycardia) is relatively trivial. The more serious adverse effect of impaired neurodevelopment was shown in a *post hoc *analysis and only applied to more mature babies. That same analysis showed that there was a possible advantage for the very immature babies, who are the subjects of this study. The neonatal community and clinicians in our unit are at equipoise concerning the value of T4 supplementation in this patient group. We will ensure that our results are suitable for meta-analysis with the work of other groups. Thus we believe that it is ethical to ask parents to allow their child to be randomised to T4 or placebo, given the monitoring that will be included in the study design.

### Regulatory bodies

EUDRACT number : 2005-003-09939

MHRA: approval granted 15^th ^June 2007

## Competing interests

The authors declare that they have no competing interests.

## Authors' contributions

SMN and AMW conceived the study, participated in its design and coordination and drafted the manuscript. MT, MD and SV participated in the design of the study. CG participated in the design of the study and the statistical analysis. All authors read and approved the final manuscript.
